# A Bump in the Road: How the Hostile AML Microenvironment Affects CAR T Cell Therapy

**DOI:** 10.3389/fonc.2020.00262

**Published:** 2020-02-28

**Authors:** Rebecca Epperly, Stephen Gottschalk, M. Paulina Velasquez

**Affiliations:** ^1^Department of Oncology, St. Jude Children's Research Hospital, Memphis, TN, United States; ^2^Department of Bone Marrow Transplantation and Cellular Therapy, St. Jude Children's Research Hospital, Memphis, TN, United States

**Keywords:** chimeric antigen receptor, cellular therapy, immunotherapy, acute myeloid leukemia, microenvironment

## Abstract

Chimeric antigen receptor (CAR) T cells targeting CD19 have been successful treating patients with relapsed/refractory B cell acute lymphoblastic leukemia (ALL) and B cell lymphomas. However, relapse after CAR T cell therapy is still a challenge. In addition, preclinical and early clinical studies targeting acute myeloid leukemia (AML) have not been as successful. This can be attributed in part to the presence of an AML microenvironment that has a dampening effect on the antitumor activity of CAR T cells. The AML microenvironment includes cellular interactions, soluble environmental factors, and structural components. Suppressive immune cells including myeloid derived suppressor cells and regulatory T cells are known to inhibit T cell function. Environmental factors contributing to T cell exhaustion, including immune checkpoints, anti-inflammatory cytokines, chemokines, and metabolic alterations, impact T cell activity, persistence, and localization. Lastly, structural factors of the bone marrow niche, secondary lymphoid organs, and extramedullary sites provide opportunities for CAR T cell evasion by AML blasts, contributing to treatment resistance and relapse. In this review we discuss the effect of the AML microenvironment on CAR T cell function. We highlight opportunities to enhance CAR T cell efficacy for AML through manipulating, targeting, and evading the anti-inflammatory leukemic microenvironment.

## Introduction

Chimeric Antigen Receptors (CARs) are a novel immunotherapeutic strategy that incorporates the antigen specificity of an antibody's single chain variable fragment (scFv) with the transmembrane and intracellular signaling domains of the CD3ζ chain and costimulatory molecules (1, 2). CD19 targeted CAR T cell therapy has proven successful for the treatment of relapsed/refractory B cell acute lymphoblastic leukemia (ALL) (3–7). Efforts to expand adoptive immunotherapy strategies to acute myeloid leukemia (AML) are complicated by antigen overlap between normal hematopoietic progenitor cells (HPCs) and leukemic blasts (8). Early clinical studies of CAR T cells for AML are ongoing, exploring several targets including CD123 (9, 10), CD33 (11), C-type lectin-like molecule 1 (CLL-1) (12, 13), and Lewis-Y (14). Additional targets under preclinical investigation include CD135 (FLT-3 receptor) (15–17), Folate receptor β (18), CD44v6 (19), WT1 (20), B7-H3 (21–23), CD70 (24, 25), and CD7 (26). While clinical experience thus far has shown feasibility and safety of CAR T cells for AML, efficacy has been limited in comparison to CD19-CAR T cell therapy for ALL (11, 14).

**Figure 1:**
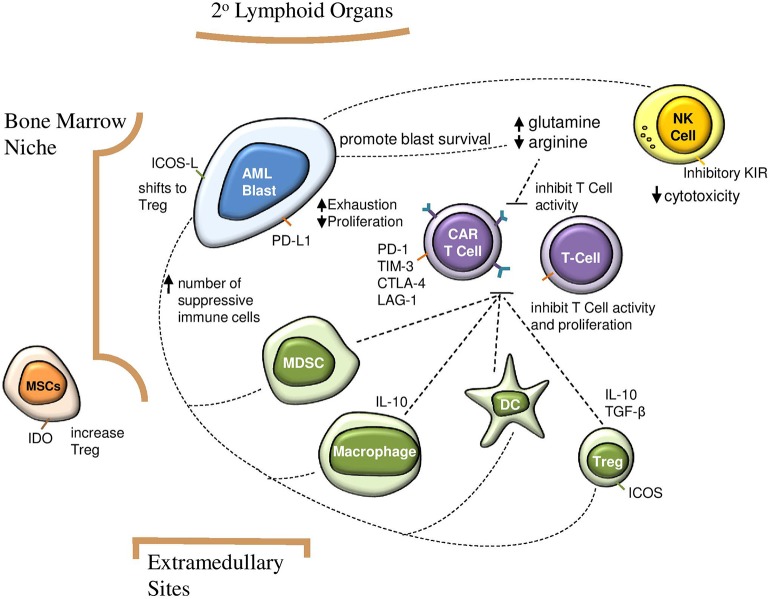
Impact of suppressive AML microenvironment on immunosurveillance and CAR T cell efficacy. AML blasts increase number of suppressive immune cells, which in turn inhibit CAR T cell activity and proliferation. Direct interactions between AML blasts and CAR T cells contribute to T cell exhaustion and decreased proliferation. A glutamine rich and arginine low environment contributes to AML blast survival and impairs CAR T cell function. AML, acute myeloid leukemia; CAR, chimeric antigen receptor; NK, natural killer; Treg, regulatory T cell; MDSC, myeloid derived suppressor cell; MSC, mesenchymal stem cell; DC, dendritic cell; IDO, indoleamine 2,3-dioxygenase; ICOS, inducible T cell costimulator.

In addition to these challenges, the AML microenvironment is highly immunosuppressive. Data on the direct impact of this aspect on CAR T cells in AML is still emerging. However, we can extrapolate from the well-established role of the microenvironment in CAR T cell therapy for solid tumors (27, 28), clinical experience with CD19-CAR T cells for B-ALL, and evidence detailing the dynamics of the AML microenvironment. This includes complex interactions between native immune cells, secreted factors, and structural components (Figure 1). In this review we discuss how each of these aspects can impact CAR T cell therapy and highlight opportunities to improve CAR T cell therapy for AML.

## Cellular Interactions

The AML microenvironment contains cell types which can dampen T cell responses. These include AML blasts, myeloid-derived suppressor cells (MDSCs), regulatory T cells (Tregs), macrophages, and dendritic cells. Counteracting these cellular interactions which promote leukemic survival may bolster the efficacy of CAR T cell therapy in AML.

### AML Blasts

In order to escape immune surveillance, AML blasts downregulate major histocompatibility complex (MHC) class I and II expression (29–32) and express inhibitory ligands such as programmed death-ligand 1(PDL-1), B7-H3 (CD276), and Galectin 9 (Gal-9) (33–35). Immune checkpoint ligands expressed on cells in the AML microenvironment interact with receptors including programmed death receptor 1 (PD-1), cytotoxic T-lymphocyte associated protein 4 (CTLA-4), lymphocyte-activation gene 3 (LAG3), and T cell immunoglobulin and mucin-containing-3 (TIM3) on T cells, leading to T cell exhaustion (34, 36, 37). Despite success of antibody-based immune checkpoint blockade for solid tumors, only modest response has been demonstrated in early trials for AML (38–40). While the mechanism for limited efficacy of immune checkpoint inhibitors as monotherapy in AML is not well-understood, disease burden is most likely one contributing factor. Thus, there is still potential for application of checkpoint inhibitors as combination therapies. In the case of combination with CAR T cells, there is the additional potential benefit that checkpoint inhibitors may improve T cell persistence and enhance response.

CAR T cells have been designed to intrinsically block PD-1 through secreted single chain variable fragments (scFv) or antibodies, shRNA, dominant-negative receptors, and CRISPR/cas9 mediated knockout (41–44). Intrinsically blocking PD-1 has similar preclinical efficacy in preventing PD-1 mediated T cell exhaustion as co-administration of PD-1/PDL-1 antibodies, with the added benefit of localizing the effect to the site of T cell activity.

Additionally, AML blasts release reactive oxygen species and indolamine 2, 3 dioxygenase (IDO). IDO in turn catabolizes tryptophan degradation, which interferes with T cell proliferation and effector function (45, 46). Leukemic blasts recruit other immunosuppressive cells such as MDSCs and Tregs to the tumor microenvironment, as well as altering metabolite availability.

### Myeloid-Derived Suppressor Cells

MDSCs are immature myeloid cells that arise in the bone marrow from myeloid progenitors (47, 48). Their presence has been documented in cancer patients regardless of tumor, and their potent immunosuppressive function is widely recognized (49). An increase in inflammatory mediators including interleukin (IL) 6, IL1β, granulocyte-macrophage colony-stimulating factor (GMCSF) and granulocyte-colony stimulating factor (GCSF) drive the accumulation and inhibitory function of MDSCs. MDSCs localize to the tumor microenvironment through chemokines such as VEGF (50). MDSCs polarize macrophages, inhibit natural killer (NK) cell-mediated tumor cell lysis and recruit regulatory T cells (Tregs). They prevent T cell activation by sequestering essential amino acids, generating reactive oxygen species (ROS), and downregulating the expression of CD62L on circulating T cells (51, 52).

An expansion in the number of MDSCs has been reported in AML patients, where they inhibit T cell responses (53–56). Expansion of MDSC decreases CAR T cell efficacy in solid tumors (57), and interventions that aim to inhibit MDSC function have had preclinical success in improving CAR T cell function (58). Given this inhibitory role on T cell proliferation and activity, MDSCs have the potential to contribute to resistance to CAR T cell therapy. Thus, strategies that are specific for antigens such as CD33 which are also present on MDSCs have antitumor activity not only through T-cell mediated direct cytotoxicity of CD33+ blasts, but also through inhibition of CD33+ MDSC (55). This is the case for a CD33xCD3 bispecific T-cell engaging antibody (BiTE)^55^ and potentially of CD33-CAR T cells (59–61).

### Regulatory T Cells

Tregs limit activation and proliferation of cytolytic T cells. They act both directly through anti-inflammatory cytokine secretion and contact dependent suppression and indirectly, by interfering with the activation status of antigen presenting cells (62). The AML microenvironment favors the expansion of Tregs (37, 63–65). Expression of inducible T cell co-stimulator ligand (ICOSL) on AML blasts stimulates T cells through inducible T cell co-stimulator (ICOS), leading to differentiation to Treg phenotype and expansion of the Treg subset (66). Additionally, AML blasts and bone marrow mesenchymal cells overexpress IDO, which promotes the emergence of a Treg phenotype and limits T cell proliferation (67).

The depletion of Tregs in a murine AML model led to an increase in proliferation and activity of adoptively transferred tumor reactive cytotoxic T cells, highlighting the immunosuppressive capabilities of Tregs in the AML microenvironment (68). In the clinical setting a lower frequency of Tregs has proven predictive of better antitumor response to the CD19xCD3 bispecific BiTE blinatumomab for ALL (69).

Several strategies can potentially circumvent the inhibitory effects of Tregs on CAR T cells. The integration of co-stimulatory domains in second or third generation CAR T cells allows CAR T cells to proliferate despite the inhibitory effects of Treg cells (70). Genetic modifications in the PYAP Lck-binding motif of CD28 costimulatory domain of a CD28.4-1BB CAR have resulted in disruption of the IL2 signaling pathway, blocking Treg activity with modest enhancement of efficacy in preclinical solid tumor models (71). In addition, transgenic expression of IL15 in CAR T cells favors proliferation of cytotoxic T cells over Tregs (72). Additionally, the administration of lymphodepleting chemotherapy prior to CAR T cell infusion depletes Tregs in the tumor microenvironment and allows for expansion of adoptively transferred CAR T cells (71).

### Natural Killer Cells

NK cells that are present in the AML microenvironment are often dysfunctional since it promotes the expression of inhibitory Killer-Cell Immunoglobulin-like Receptors (KIRs) resulting in decreased interferon (IFN)-γ secretion and cytolytic capacity (73). Additionally, downregulation of micro-RNAs (MIRs), single stranded non-coding RNAs that play a role in gene expression, leads to a downregulation of IL2 and IL15 cytokine receptors, NKG2D, and transcription factors such as c-myb in NK cells (74–76). Downregulation of transcription factors inhibits the activity of naturally occurring NK cells in the leukemic microenvironment. Despite this, the infusion of unmodified donor-derived NK cells in post-transplant patients has proven beneficial in controlling leukemia relapse by contributing to Graft-vs.-Leukemia (GVL) effects (77–81). This antitumor response can be enhanced by generation of a cytokine-induced memory-like NK cell subset (82). Genetic modifications to NK cells including introduction of CARs have been investigated as a platform for cellular immunotherapy in preclinical leukemia models (83–86). CD19-CAR NK cells expressing IL15 have shown improved persistence, with encouraging results in an ongoing clinical study (NCT03056339) (86). CAR NK cells have potential benefit for use as an allogeneic product given a lower risk of graft-vs.-host disease than T cell-based therapies as well as the presence of native activating receptors such as NKG2D, which may amplify antitumor activity (77, 87–89).

### Myeloid Cells

Macrophages can perform both inhibitory and stimulatory functions. In the AML microenvironment, blasts and MDSCs skew macrophage differentiation to an inhibitory phenotype (90). Inhibitory macrophages contribute to a hostile environment for CAR T cells in AML. In patients receiving CD19-CAR T cells for B cell lymphoma, infiltration with tumor associated macrophages diminishes response to CAR T cells (91). Macrophages have been implicated in the pathogenesis of cytokine release syndrome associated with CAR T cell therapy (92), evidence that modulation of macrophage function has significance for titrating clinical response.

Dendritic cells also play an important role in modulating the immune microenvironment. AML blasts can cause arrest of dendritic cell maturation, promoting immune tolerance and inducing Treg development (93). Chemotherapy induced cell death contributes to production of tolerogenic dendritic cells, potentially impacting immunotherapy efforts when used in combination (94). Driving dendritic cells toward a T cell stimulatory phenotype could improve CAR T cell therapy. For example, CAR T cells genetically modified to constitutively express CD40 ligand promote secretion of the pro-inflammatory cytokine IL12 by dendritic cells, which enhances antitumor efficacy (95).

## Soluble Environmental Factors

Many suppressive effects of the AML microenvironment are mediated through soluble environmental factors. AML blasts influence cells in the microenvironment to secrete anti-inflammatory cytokines and alter chemokine-mediated trafficking. Metabolic changes drive the microenvironment to support leukemic cell growth and survival while limiting immune responses.

### Anti-inflammatory Cytokines and Chemokines

Both exogenous and endogenous cytokines are integral in promoting the expansion and effector function of CAR T cells (96, 97). Specific cytokine signatures have been linked to efficacy, for example increased IL6 levels observed in responders to CD19-directed CAR T therapy for ALL (98). While the presence of AML blasts can stimulate monocytes to secrete pro-inflammatory cytokines including tumor necrosis factor-α (TNF-α), IL1β, and IL6, they also cause increased production of the anti-inflammatory cytokine IL10 (93). Additionally, increased Treg subsets contribute to production of IL10 and transforming growth factor β (TGF-β), which can limit the effector function of CAR T cells (66). In analysis of CD123-CAR T cells targeting AML, TNF-α and IFN-γ upregulate CD123 expression on endothelial cells, increasing risk for capillary leak. Production of IL6 and IL1 by monocytes and macrophages mediates cytokine release syndrome and is associated with neurotoxicity in the CD19-CAR clinical experience (99, 100). This illustrates that the cytokine milieu in the leukemic microenvironment impacts not only antitumor activity of CAR T cells, but also toxicities.

Chemokines play an important role in trafficking of T cells to the lymphoid compartment and toward malignant cells in other sites. The serum chemokine profile in AML patients differs from healthy controls including levels of CCL3, CCL4, CCL5, CCL17, and CXCL10 (101, 102). Variations in systemic chemokine levels and expression of chemokine receptors in patients with AML have been linked with prognosis and treatment response (103, 104). Chemokine-mediated trafficking has been exploited to enhance CAR T cell activity. For example, forced co-expression of the chemokine receptor CCR4 with CAR increases accumulation of CD8+ effector CAR T cells in the lymphoid compartment in a Hodgkin Lymphoma model (105). Expression of chemokine ligand CCL19 together with IL7, which are typically produced by lymphoid T-cell zones, enhances CAR T infiltration into solid tumors (106). These strategies are particularly relevant to targeting CAR T cells toward AML disease burden in chloromas or extramedullary sites.

### Metabolic Alterations

The immunosuppressive capabilities of the AML microenvironment are potentiated by metabolic alterations including changes in amino acid and nucleotide concentrations. The AML microenvironment rich in glutamine contributes to immunosuppression as higher concentrations of this amino acid inhibit T cells by contributing to T cell exhaustion (107). Inhibiting glutamine metabolism using L-asparaginase, a chemotherapeutic agent that also has glutaminase activity, has proven effective treating AML (108–111). In addition, culturing T cells in glutamine restricted media can improve antitumor activity of CD8+ T cells by reducing T cell exhaustion and allowing for enrichment of an effector memory T cell subset (107). Thus, modulating glutamine concentrations in culture media has the potential to improve CAR T cell activity in a similar fashion.

Conversely, AML blasts promote a low arginine microenvironment mediated through the expression of arginase II. Limiting arginine availability steers monocytes toward a suppressive phenotype and acts as a metabolic brake for T cells, evidenced by lower IFN-γ production and increased expression of checkpoint inhibitors leading to decreased proliferation (112). Inhibiting arginine metabolism enhances antitumor activity of CD33-CAR T cells for AML in preclinical studies (113), demonstrating the importance of metabolic dynamics on efficacy of CAR T cell therapy.

Increases in adenosine concentration also inhibit T cell activity in the leukemia microenvironment. Adenosine is metabolized by CD73 and CD39 from extracellular ATP (114, 115). CD73 is expressed on tumor cells, MDSCs and Tregs (114), while CD39 has been described on CD8+ T cells. Increased adenosine leads to signaling through adenosine receptors, such as adenosine 2A (A2A), which in turn results in T cell suppression. Altering adenosine metabolism by targeting CD73 or CD39 with monoclonal antibodies or inhibitory drugs result in more effective antitumor activity (116). Targeting downstream adenosine metabolism by blocking A2A receptors with pharmacological agents has resulted in enhanced CAR T cell therapy for solid tumors preclinically (114), but its role in hematologic malignancies is less established.

## Structural Components

The physical spaces where AML blasts reside include the bone marrow niche, secondary lymphoid organs, and extramedullary sites. For CAR T cells to be effective, they not only have to penetrate the complex bone marrow environment, but also sanctuary sites such as the central nervous system, which can harbor blasts and allow for immune escape.

### Bone Marrow Niche

The bone marrow microenvironment includes hematopoietic, endothelial, osteoblastic, and stromal components. Mesenchymal stem cells (MSCs) are stromal cells which define the bone marrow microenvironment and give rise to other supporting cells. MSCs highly express IDO, which correlates with expansion of Tregs and could inhibit CAR T cell effector function (117).

MSCs from AML patients have a higher propensity to differentiate into adipocytes, and the interactions between AML blasts and adipocytes in the bone marrow niche impact cellular metabolism (118). AML blasts induce lipolysis in bone marrow adipocytes, shifting toward fatty acid oxidation and an environment favorable for leukemic survival (119). These AML-adipocyte interactions have been linked to chemotherapeutic resistance (120, 121). The ability of substrates for fatty acid oxidation can impact not only AML blast survival, but also T cell persistence. Specifically, fatty acid oxidation is key in development of memory CD8+ T cells (122, 123). Signaling through a 4-1BB costimulatory domain is associated with a shift toward fatty acid oxidation rather than glycolysis (124), which is proposed as a potential mechanism for improved persistence of 41-BB containing CAR constructs (124).

Interactions through the chemokine receptor CXCR4/CXCL12 pathway are integral in leukocyte trafficking in the bone marrow niche, involving both the endothelium and leukocytes. CXCR4 expression dictates AML blast migration, is implicated in prognosis, and is being explored as a therapeutic target (125). The CXCR4 pathway can also be involved in migration of CAR T cells to the bone marrow niche, demonstrated by improvement in bone marrow localization when CAR T cells are co-transduced with CXCR4 in preclinical studies (126).

Inflammatory responses impact the interaction between HPCs and the bone marrow niche, prompting quiescent HPCs to actively proliferate. This has been demonstrated secondary to viral infections (127), chemotherapy, and mediated through cytokines including the IFN-α signaling pathway (128). This IFN-α based shift in HSC populations can sensitize leukemic stem cells to cytotoxic therapies and has justified the use of IFN-α in management of chronic myelogenous leukemia. IFN-α supports proliferation of T cells, and co-administration of IFN-α with CD19-CAR T cells has shown enhanced activity *in vitro* for treatment of B cell lymphoma (129). The impact on myeloid progenitors in the bone marrow niche and enhanced T cell proliferation suggests a potential benefit for combining IFN-α with CAR T cell therapy to enhance anti-leukemic effect in AML.

### Secondary Lymphoid Organs

Clinical trials with CD30-CAR T cells in Hodgkin lymphoma and CD19-CAR T cells in non-Hodgkin lymphoma have shown that CAR T cells do penetrate into lymph nodes and have persistent antitumor activity (130, 131). While lymphoid tissues have an important role to enhance antigen presentation and selective T cell proliferation, fibroblastic reticular cells (FRC) can attenuate T cell expansion through immune suppressive mediators including IDO, A2A receptor, prostaglandins, and TGFβ (132, 133). This suppressive effect has been demonstrated on native T cells both in murine models and humanized *in vitro* systems, however there is some evidence that activated effector CAR T cells may be resistant to this suppression (133).

### Extramedullary Sites

AML demonstrates a variety of extramedullary manifestations, either in isolation or associated with bone marrow disease (134, 135). Chloromas are noted both at the time of initial diagnosis and relapse. The central nervous system and reproductive organs are particularly vulnerable to relapse, including after allogeneic hematopoietic stem cell transplant, as they can act as sanctuary sites to harbor leukemic cells through physical barriers (136). In order for CAR T cell therapy to be effective in treating refractory or relapsed AML, CAR T cells must be able to penetrate and persist in these sites. In clinical studies, CD19-CAR T cells have been shown to infiltrate, expand, and have antitumor activity in the CNS (137) and reproductive sites (138).

## Conclusion

The hostile AML microenvironment has a notable role in dampening T cell effector function. The cellular interactions, soluble environmental factors, and structural components of the AML microenvironment have potential to limit antitumor efficacy of CAR T cells. Investigating complex interactions between the AML microenvironment, CAR T cell therapy, and other novel anti-leukemic therapies allows the opportunity to improve upon our current regimens. Targeting antigens shared between AML blasts and suppressive immune cells such as CD33 and B7-H3 present the opportunity to modulate the microenvironment while targeting tumor cells. Designing CAR T cells capable of modulating the microenvironment's cytokine and chemokine milieu have the potential to enhance T cell effector function, leading to increased antileukemic activity. In addition, exploring combinatorial therapies with antibodies and other pharmacological compounds, such as checkpoint inhibitors or adenosine receptor blockers may improve CAR T cell efficacy and persistence. In our opinion, incorporation of combination therapies would tackle antigen escape and bypass limitations regarding the number of additional CAR modifications that can be performed with current technologies. Current clinical experience has stemmed predominantly from autologous CAR T cells. The use of allogeneic CAR T cells could overcome limitations of autologous T cell production including logistics and reduced T cell quality in heavily pretreated patients. However, most allogeneic CAR T cell products require additional genetic engineering to reduce the risk for graft-vs.-host effect; in addition their *in vivo* expansion and persistence may be limited in comparison to autologous products. As we gain insights into the intricate dynamics that affect modulation of immune cells, there is an opportunity to convert an immunosuppressive microenvironment into one that favors CAR T cell effector function and persistence.

## Author Contributions

RE and MV conceptualized the manuscript. RE, SG, and MV provided content. All authors reviewed, edited, and approved the final manuscript.

### Conflict of Interest

SG and MV hold patent applications in the field of gene and cell therapy. The remaining author declares that the research was conducted in the absence of any commercial or financial relationships that could be construed as a potential conflict of interest.
